# Synthesis of Nanofibrillated Cellulose by Combined Ammonium Persulphate Treatment with Ultrasound and Mechanical Processing

**DOI:** 10.3390/nano8090640

**Published:** 2018-08-21

**Authors:** Inese Filipova, Velta Fridrihsone, Ugis Cabulis, Agris Berzins

**Affiliations:** 1 Latvian State Institute of Wood Chemistry, Dzerbenes Street 27, LV-2121 Riga, Latvia; fridrihsone.velta@inbox.lv (V.F.); cabulis@edi.lv (U.C.); 2 Faculty of Chemistry, University of Latvia, Jelgavas Street 1, LV-1004 Riga, Latvia; agris.berzins@lu.lv

**Keywords:** nanocellulose, ammonium persulfate, oxidation, nanofibrils, high shear mixer

## Abstract

Ammonium persulfate has been known as an agent for obtaining nanocellulose in recent years, however most research has focused on producing cellulose nanocrystals. A lack of research about combined ammonium persulfate oxidation and common mechanical treatment in order to obtain cellulose nanofibrils has been identified. The objective of this research was to obtain and investigate carboxylated cellulose nanofibrils produced by ammonium persulfate oxidation combined with ultrasonic and mechanical treatment. Light microscopy, atomic force microscopy (AFM), powder X-Ray diffraction (PXRD), Fourier-transform infrared spectroscopy (FTIR), thermogravimetric analysis (TGA) and Zeta potential measurements were applied during this research. The carboxylated cellulose suspension of different fractions including nanofibrils, microfibrils and bundles were produced from bleached birch Kraft pulp fibers using chemical pretreatment with ammonium persulfate solution and further defibrillation using consequent mechanical treatment in a high shear laboratory mixer and ultrasonication. The characteristics of the obtained nanofibrils were: diameter 20–300 nm, crystallinity index 74.3%, Zeta potential −26.9 ± 1.8 mV, clear FTIR peak at 1740 cm^−1^ indicating the C=O stretching vibrations, and lower thermostability in comparison to the Kraft pulp was observed. The proposed method can be used to produce cellulose nanofibrils with defined crystallinity.

## 1. Introduction

Cellulose is a natural, linear, renewable biopolymer, composed of d-glucopyranose units, and it is present naturally in all plants on earth. Nanocellulose is material obtained by the disintegration of cellulose into nanoscale particles such as cellulose nanowhiskers (CNC), cellulose nanofibers (CNF), cellulose nanospheres (CNS) and amorphous nanocellulose (ANC) [1]. The above-mentioned nanoscale particles are extensively investigated for a wide range of different uses in eco-friendly advanced applications and materials, such as nanocomposites, bionanomaterials and others [2,3,4,5,6]. The shape of the cellulose nanoscale particles depends both on the source [7] and method of production [8]. CNC are obtained mainly by acid hydrolysis of cellulose fibers [9], whereas CNF are usually extracted by mechanical disintegration of cellulose fiber combined with biological and/or chemical pre-treatment [10]. CNF, also known as nanofibrillated cellulose, microfibrillated cellulose (MFC) and cellulose nanofibrils, is involved in a large number of advanced applications, such as nanocomposites [11,12], foams, aerogels [13], packaging [14] and others.

CNF have a high surface area covered with hydroxyl groups, which provide options for surface chemical modification to differentiate between the properties and characteristics of the target material. A large number of modification methods have been investigated in recent years, such as esterification [15], etherification, silylation, urethanization, amidation [16], click reactions [17], and polymer grafting [18]. Oxidation is known as the most commonly used method to convert cellulose into value-added derivatives [19]. Isogai et al. [20] suggested 2,2,6,6,-tetramethylpiperidine-1-oxyl radical (TEMPO)-oxidation as a simultaneous process for CNF production and its functionalization. The above-mentioned method is now widely used as a pre-treatment in order to produce carboxylated CNF for the subsequent use in a wide range of applications [21,22,23,24,25]. The disadvantage of this method is that TEMPO is very expensive and toxic.

Ammonium persulfate (APS) is known as a strong oxidizer with low long-term toxicity, high water solubility and low cost [26]. The persulfate ion S_2_O_8_^2−^ can be activated using heat or light, and the resulting SO_4_•^−^ initiates a chain of reactions involving other radicals and oxidants [27]. The first paper about the use of APS for the production of nanocellulose was published by Leung et al. [26], where a one-step procedure for producing high crystalline carboxylated CNC from different cellulosic materials was described. Currently, APS oxidation is mentioned as one of the promising oxidation methods to produce CNC [28]. APS oxidation has the potential to be used for carboxylated CNC production as an alternative to TEMPO-mediated oxidation [29]. The main advantage of the APS method is that there is no need for purification of the raw material before nanocellulose production. Since 2011, the APS method has become widely used and a number of articles about obtaining of CNC by the APS method from different feedstock, such as cotton linter [26,30,31,32,33], flax [26,34], hemp [26,34], triticate [26], micro crystalline cellulose [26,33,34,35,36], wood pulp [26,30,32,37,38], bacterial cellulose [26,39,40], borer powder of bamboo [41], cornhob [42], Lyocell [43], oil palm empty fruit bunch [44], coconut fiber [45], and sugarcane bagasse [29,46], have been published.

Most of the articles are focused on the production of carboxylated CNC by heating the cellulose materials at 60–90 °C with 1–1.5 M APS solution for 8–24 h. As a result, CNC with a diameter of 3–100 nm and length between 100 and 500 nm are obtained. CNS were obtained from sugarcane bagasse using 2 M APS solution for 16 h at 60 °C [29], from bamboo powder using 2 M APS solution for 6 h at 65 °C [33] and from Lyocell fibers using 1 M APS solution at 70 °C with various oxidation times [43]. It was reported by Goh et al. that MFC was obtained from oil palm empty fruit bunch using APS oxidation combined with ultrasonication [44].

A lack of research about combined APS oxidation and common mechanical treatment has been identified. It was reasonable to put forward a hypothesis about obtaining oxidized CNF by combining APS treatment with ultrasound and rather mild mechanical processing. Therefore, the objective of the current research was to obtain and investigate carboxylated CNF produced by APS oxidation combined with ultrasonic and mechanical treatment.

## 2. Materials and Methods

### 2.1. Chemical Treatment of Cellulose

Ten grams (dry weight) of total chlorine free bleached birch Kraft pulp (obtained from Sodra Cell AB, Sweden as dry pulp sheets) was soaked in distilled water (500 mL) for 8 h and then disintegrated to 75,000 revolutions in the Disintegrator (Frank PTI, Laakirchen, Austria). The excess water was drained using a Büchner funnel, and the pulp was repeatedly mixed with fresh distilled water (300 mL) in glass beaker. The beaker was covered in foil and the suspension was heated to 70 °C in a water bath. Afterwards, APS (purity, ≥98%; 114 g), HCl (concentration 37%; 20.53 mL) and distilled water were added to reach a final volume of 500 mL. The mixture was then heated at 70 °C for 4 h with continuous stirring. The reaction was stopped by cooling the mixture in an ice bath until it reached ~15 °C, and then oxidized cellulose was filtered using a Büchner funnel and washed until it reached the pH of distilled water.

### 2.2. Mechanical Treatment of Cellulose

The mechanical treatment of cellulose fibers was performed in a two-step procedure. First, 400 mL of oxidized cellulose slurry (cellulose content 2 wt %) was treated in high shear laboratory mixer, Silverson L5M-A (Silverson Machines, Inc., East Longmeadow, MA, USA), at 10,000 rpm for 20 min. The working head of the mixer was completely immersed in the slurry about 1 cm from the bottom of the beaker. Mechanical treatment was followed by ultrasound treatment by an ultrasonic homogenizer, SONIC-650W (MRC Ltd., Holon, Israel), for 8 min (probe diameter 10 mm, 95% of power, 25 Hz, 9 s on, 1 s off). The beaker with the slurry was cooled in the ice bath during the treatment. The combined processing cycle of the sample was repeated seven times until the viscosity visually increased, indicating the occurrence of nanocellulose.

### 2.3. Characterisation of Nanocellulose

A drop (0.04 µL) of diluted suspension (0.001 wt %) was placed on a microscope slide and allowed to dry at room temperature. Then, atomic force microscopy (AFM) measurements were performed in the tapping mode using the Park NX10 (Park Systems Corporation, Suwon, Korea). The size of the objects in the AFM pictures were measured using XEI 1.7.6. software (Park Systems Corp., Santa Clara, CA, USA).

A drop (0.5 mL) of diluted suspension (0.05 wt %) was placed on a microscope slide, covered with a coverslip and investigated by a Leica DMLB microscope connected to a Leica DFC490 video camera (both from Leica Microsystems GmbH, Wetzlar, Germany) with a magnification of 200–1000×.

For other analyses, the CNF suspension was freeze-dried and milled in a MM200 ball mill (Retsch, Haan, Germany) for 10 min at a frequency of 30 Hz.

The milled sample was mixed with KBr powder and pressed into a small tablet. From this, FTIR spectrum values were recorded using a Nicolet iS50 spectrometer (Thermo Fisher Scientific, Waltham, MA, USA) at a resolution of 4 cm^−1^, 32 scans per sample.

The thermal stability of APS CNF was compared with Kraft pulp by a TGA SDTA850e thermal analyzer (Mettler Toledo, Columbus, OH, USA) under nitrogen atmosphere, heating rate of 10 °C/min, and temperature ranging from 25 °C to 800 °C; the test sample was approximately 8–10 mg.

The powder X-ray diffraction (PXRD) patterns were measured on a D8 Advance diffractometer (Bruker, Karlsruhe, Germany) using copper radiation at a wavelength of 1.5418 Å, equipped with a LynxEye position sensitive detector. The tube voltage and current were set to 40 kV and 40 mA. The divergence slit was set at 0.6 mm and the anti-scatter slit was set at 8.0 mm. The diffraction patterns were recorded using a 1.0 s/0.02° scanning speed from 5° to 70° on a 2θ scale. Crystallinity of the cellulose sample was calculated using Rietveld refinement on the obtained PXRD patterns in TOPAS v5 (Bruker, Karlsruhe, Germany). The crystalline phase was modeled using crystallographic data of cellulose Iβ (Cambridge Structural Database (CSD) reference code JINROO01). The amorphous phase was modeled according to the partial or no known crystal structures (PONKCS) [47] approach using artificial phase with the PXRD pattern corresponding to amorphous cellulose (prepared by grinding Avicell crystalline cellulose in ball mills for 90 min in stainless steel 35 mL grinding jars with a 20 mm ball at a frequency of 15 Hz), with the calibration constant determined from a measurement of its mixture with corundum. In Rietveld refinement, the background was modeled with the Chebyshev polynomial function of second degree. For crystalline cellulose lattice parameters and crystallite size, the parameters were refined by additionally modeling the preferred orientation with spherical harmonics of sixth order.

The zeta potential was determined on Zeta Sizer Nano ZS90 (Malvern Panalytical Ltd., Malvern, UK) for 0.05 wt % CNF suspension in distilled water.

## 3. Results and Discussion

### 3.1. Microscopy

**Figure 1:**
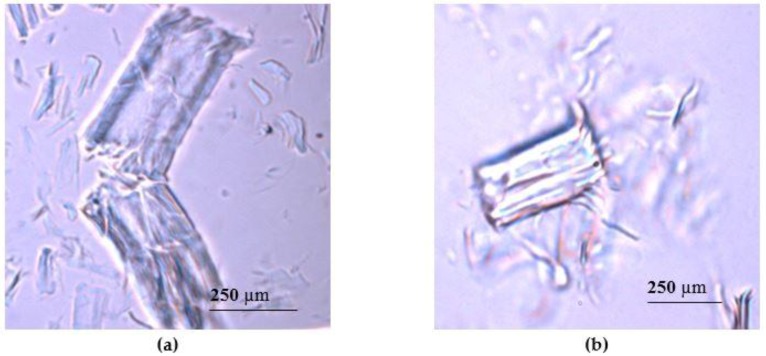
Bleached birch Kraft pulp fibers in an optical microscope (**a**) after ammonium persulfate (APS) treatment; (**b**) after APS treatment followed by three cycles of mechanical treatment in a high shear mixer and ultrasound treatment.

The synthesis of CNF started with the chemical treatment of bleached birch Kraft pulp fibers with APS solution. Structural damages were identified on the surface of fibers after chemical treatment by APS (Figure 1a). There were visible defects in certain fiber sections leading to splitting of the fiber perpendicular to the longitudinal axis. It is well known that the most accessible regions of the cellulose are disordered amorphous regions [1]; therefore, it is clear that the damages observed in Figure 1 are rather related to the destruction of cellulose polymer chains mostly in amorphous regions. The images of APS treated cellulose demonstrated a mixture of particles with sizes in range from 50 µm to 500 µm. The reaction yield was 80 ± 2% from initial pulp material.

The reaction of cellulose and APS took place when persulfate ions S_2_O_8_^2−^ were activated by the heating of the APS-cellulose solution to 70 °C in acidic conditions. Thus, SO_4_•^−^ was created in the mixture according to the previously reported reaction mechanism [27,48]. The proposed further reaction course involves the presence of other radicals and oxidants, including hydrogen peroxide. Both hydrogen peroxide and radicals penetrate the amorphous regions of cellulose and break down the disordered cellulose by co-hydrolyzing the 1,4-β bonds of the cellulose polymer chain [26], causing the observed loss of cellulose mass. Almost all authors who have worked with this method previously reported depolymerization of cellulose by APS treatment [26,29,30,31,33,35,38,39,41,43,44,45,46,49,50,51].

It was found that the depolymerization process of cellulose depends on the concentration of APS solution, treatment time [43] and mainly on the type of cellulose source [26]. The majority of published research on APS treatment of cellulose materials have focused on the total destruction of amorphous regions in order to obtain CNC. This research was devoted to the partial destruction of amorphous cellulose; therefore, a shorter reaction time was chosen in order to partly preserve the natural structure of cellulose. The partly depolymerized cellulose was used as a raw material for CNF production employing mechanical treatment.

**Figure 2:**
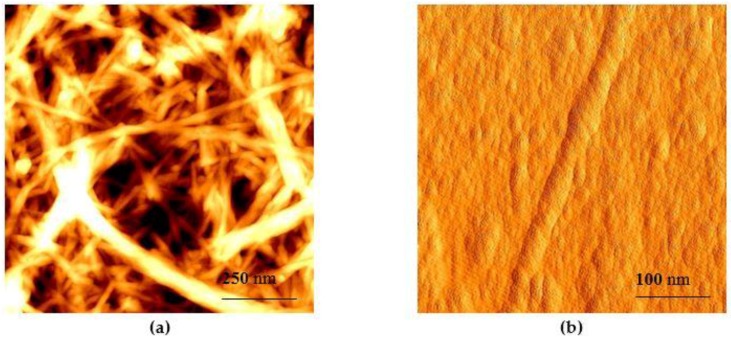
Atomic force microscopy (AFM) pictures of cellulose nanofibers (CNF) obtained from bleached birch Kraft pulp by combined chemical, mechanical and ultrasound treatment (**a**) a bundle of CNF; (**b**) a single fiber of CNF.

The processing of cellulose was continued by seven cycles of the mechanical and ultrasound treatment. The ultrasound treatment was chosen as an intermediate step between the mechanical treatments in a high shear mixer to create additional hydrodynamic shear forces, which can contribute to the delamination process of cellulose fiber. MFC production by APS oxidation combined with ultrasonication was reported previously by Goh et al. [44]. The visualization of material after APS oxidation and mechanical treatments (CNF obtained by APS and mechanical treatment, hereinafter APS CNF) demonstrated delamination of cellulose fiber (Figure 1b). It was observed that after three cycles of mechanical treatment, individual micro- and nanofibrils can be seen separating from the bulk fiber. The level of fibrillation increased when the number of the treatment cycles was increased, leading to the high level of fibrillation seen after seven cycles of the treatment. The occurrence of CNF was identified by the increase in the apparent viscosity of the cellulose mixture and by AFM imaging. It was found that the obtained material consisted of a micro- and nanofibril mixture (Figure 2a), and objects with diameters in range from 20 nm to 300 nm were identified (Figure 2b). It is known that the presence of CNF can be proved by observing cellulose with lateral dimensions in the nanometer range [10]. This is also in accordance with the typical diameters of CNF reported by other authors [44]. The length of the fibrils was not detectable because of the entanglements. The presence of micro and macro objects in CNF correlates to the statement of Desmaisons et al., who reported that despite the fact that a CNF suspension in reality is composed of nano, micro and macro dimensions caused by incomplete defibrillation during mechanical shearing, it still can meet the quality standards of CNF [52].

It is known that obtaining CNF is a high energy demanding process because of the use of high-pressure homogenizers, microfluidizers, grinders etc. [10]. The high energy demand has been a challenge for decades and the main barrier for commercial success until the strong impact of pretreatment methods was discovered, such as TEMPO oxidation or enzymatic hydrolysis. It has been identified that more focus should be devoted to the development of alternative pretreatment methods, which can benefit the production process or endow the CNF with new properties [10]. The goal of this research agrees with the previous statement because APS oxidation is used as a pretreatment method prior to the mechanical treatment of cellulose. A similar approach, with an oxidation step before rather mild mechanical treatment, has been already used before. It was reported that it is possible to obtain CNF by blending 0.7–2.0 wt % oxidized cellulose suspensions with conventional blenders [53,54].

### 3.2. FTIR Results

**Figure 3:**
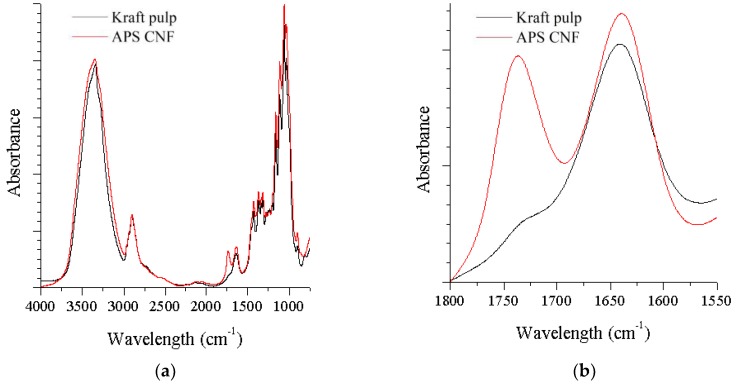
FTIR spectra of ammonium persulfate treated nanofibrillated cellulose compared to microcrystalline cellulose (**a**) spectra 4000–850 cm^−1^; (**b**) spectra 1800–1550 cm^−1^.

The FTIR spectra of neat cellulose and APS CNF are presented in Figure 3a. For both samples, the peak observed at ~3400 cm^−1^ is attributed to O–H vibration, mainly caused by hydrogen bonds in the cellulose. The peak at ~2900 cm^−1^ is associated with C–H stretching vibrations, and the peak at ~1640 cm^−1^ is related to the absorbed water. The peak at ~1375 cm^−1^ is associated with asymmetric vibrations of C–H band; and sharp peaks at 1060 cm^−1^ are associated with C–O stretching vibrations. Both cellulose and APS CNF exhibited similar peaks (Figure 3a), with exception of a peak at 1740 cm^−1^ for APS CNF (Figure 3b), which is related to the C=O stretching vibration. The presence of C=O confirmed that the hydroxyl groups of the CNF had been oxidized to carboxyl groups during APS treatment. Results correlate with other authors [38,51] who reported that the hydroxyl groups at C6 of cellulose were regioselectively oxidized into carbonyl groups during APS treatment. The charges on the oxidized cellulose fibers enhance the swelling of the cellulose by electrostatic repulsion to facilitate the fibrillation into nanofibers [20]. Furthermore, it was reported that the presence of carbonyl groups on the surface of cellulose nanoparticles would make them more reactive and improve their properties needed for their use in nanocomposites [43], or make them more accessible for chemical derivatization, such as grafting [55].

### 3.3. Thermal Stability

**Figure 4:**
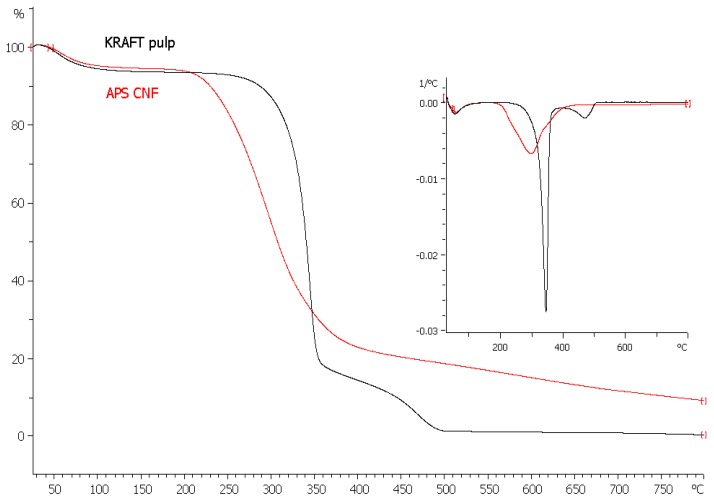
TG (big scale) and differential thermogravimetry DTG (small scale) curves of Kraft pulp and ammonium persulfate treated nanofibrillated cellulose (APS CNF).

The thermal stability of the obtained APS CNF was investigated by TGA analysis. The TGA and differential thermogravimetry (DTG) curves of APS CNF in comparison to initial Kraft pulp are shown in Figure 4. The weight loss at temperatures below 120 °C corresponds to the evaporation of adsorbed water [33].

Kraft pulp displayed the typical degradation profile of cellulose [56] with an onset temperature of 306 °C, while thermal degradation of APS CNF started at 225 °C, which was significantly lower than that of the initial pulp. Based on the DTG curves, the thermal decomposition peaks for the maximum weight loss of the Kraft pulp and APS CNF were at 347 °C and 298 °C, respectively. The thermal stability of cellulose material decreased after APS and mechanical treatments, most likely due to the mechanical degradation of cellulose [57]. The decreased degradation temperature of APS CNF could be related to the presence of carboxylic groups [46] and the structure of nanofibrillated cellulose, which have increased surface area compared to the Kraft pulp. Results were in good accordance to other authors [29,33], who reported about lower thermal stability of nanocellulose obtained by ammonium persulfate oxidation compared to the initial cellulosic material.

### 3.4. PXRD Crystallinity

**Figure 5:**
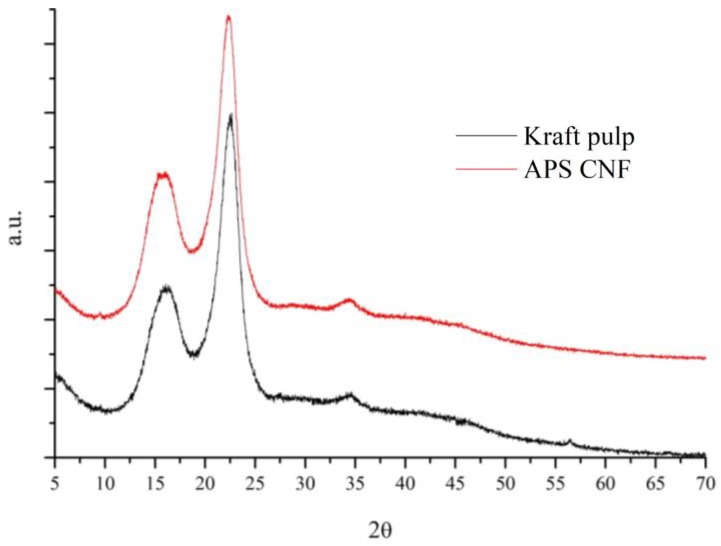
Powder X-ray diffraction spectra of persulfate treated nanofibrillated cellulose (APS CNF) and Kraft pulp.

The powder X-ray diffraction studies were conducted to estimate the crystallinity of obtained CNF. Figure 5 gives the PXRD spectra of APS CNF compared to the initial bleached Kraft pulp. As can be observed, both samples showed three characteristic peaks at 16°, 22.7° and 34.5°, representing the (110), (200) and (004) crystallographic planes of the monoclinic cellulose I lattice [58]. Crystallinity of the cellulose sample was calculated using Rietveld refinement on the obtained PXRD patterns in TOPAS v5. The crystallinity index (CI) of APS CNF was 74.3%, which was higher than the CI of the initial bleached Kraft pulp (69.6%). The higher CI of APS CNF indicated that part of the amorphous region in cellulose had been removed during APS treatment. A similar correlation was also found by Goh et al. [44], where oil palm empty fruit bunches were used as a raw material for obtaining MFC. The raw material had a CI of 74%, which was increased to 75% of MFC by APS pretreatment and ultrasonication. According to the investigations of Chen et al., the ultrasonic treatment had an insignificant impact on the crystal regions of the cellulose fibers [59]. We suggest that changes in the CI of APS CNF were made mainly by chemical pretreatment, while mechanical treatment left the CI of cellulose unchanged.

The proposed method can be used to produce CNF with a defined CI, using pretreatment with APS solution to increase the CI of cellulose and a high shear mixer to defibrillate fibers. It has been reported by Jiang et al. that APS oxidation of cellulose material is easily controllable [38]. However, the majority of researchers have used APS treatment to disrupt the amorphous regions of the cellulose to reach the highest CI in order to obtain CNC. By employing APS oxidation with a duration of 12–16 h, it has been possible to produce CNC with a CI in range from 86.8% [51], 87% [38] and up to 93.9% [43].

### 3.5. Zeta Potential

The nanocellulose suspension is considered stable when the absolute value of the Zeta potential is higher than −25 mV [60,61]. The measurements of the Zeta potential of APS CNF were carried out after each cycle of the mechanical treatment. The average number was −26.9 ± 1.8 mV. It was identified that processing time and the number of cycles had no impact on the Zeta potential. It can be concluded that the required value of Zeta potential in order to obtain a stable suspension has been achieved in case of the APS CNF.

## 4. Conclusions

An innovative method for the production of cellulose nanofibrils with a diameter 20–300 nm by ammonium persulfate and further defibrillation using consequent mechanical treatment in a high shear laboratory mixer and ultrasonication was developed. It was found that the suspension of ammonium persulfate treated cellulose nanofibrils consists of different fractions, including nanofibrils, microfibrils, bundles and others. However, the suspension was considered stable because the Zeta potential value was −26.9 ± 1.8 mV. The crystallinity index of ammonium persulfate treated nanofibrillated cellulose was 74.3%, which was higher than the crystallinity index of the initial bleached Kraft pulp (69.6%). The thermal stability of cellulose material decreased after ammonium persulfate and mechanical treatments. The proposed method can be used to produce cellulose nanofibrils with defined crystallinity, using pretreatment with ammonium persulfate solution to increase the crystallinity index of cellulose and a high shear mixer and ultrasonication to defibrillate the fibers. The homogeneity of the produced cellulose nanofibril suspension is the main challenge for this method.
